# Vitamin A depletion alters sensitivity of motor behavior to MK-801 in C57BL/6J mice

**DOI:** 10.1186/1744-9081-6-7

**Published:** 2010-01-22

**Authors:** Ming Zhang, Baohu Ji, Hong Zou, Junwei Shi, Zhao Zhang, Xingwang Li, Hui Zhu, Guoyin Feng, Meilei Jin, Lei Yu, Lin He, Chunling Wan

**Affiliations:** 1 Bio-X Center, Key Laboratory for the Genetics of Developmental and Neuropsychiatric Disorders (Ministry of Education), Shanghai Jiao Tong University, Shanghai 200030, PR China; 2 Institutes for Nutritional Sciences, Shanghai Institute of Biological Sciences, Chinese Academy of Sciences, 319 Yueyang Road, Shanghai 200031, PR China; 3 Shanghai Institute for Biological Science, Chinese Academy of Science, 500 Cao Bao Road, Shanghai 200233, PR China; 4 Shanghai Institute of Mental Health, 600 South Wan Ping Road, Shanghai, PR China; 5 Institutes of Biomedical Sciences, Fudan University, 130 Dongan Road, Shanghai 200032, PR China; 6 Department of Genetics and Center of Alcohol Studies, Rutgers University, 607 Allison Road, Piscataway, New Jersey 08854, USA

## Abstract

**Background:**

Vitamin A and its derivatives (retinoids) are crucial for the development, maintenance and morphogenesis of the central nervous system (CNS). Although motor impairment has been reported in postnatal vitamin A depletion rodents, the effect of vitamin A depletion on homeostasis maintaining capability in response to external interference is not clear.

**Methods:**

In the current study, we measured the effect of vitamin A depletion on motor ability and pain sensitivity under two different conditions: 1. prior to any injection and 2. after the injection of an N-methyl-D-aspartate (NMDA) receptor antagonist (MK-801).

**Results:**

Vitamin A depletion mice showed decreased body weight, enhanced locomotor activity, increased rearing and less tail flick latency. Vitamin A depletion also induced hypersensitivity of stereotypy, ataxia, rearing, and tail flick latency to MK-801, but hyposensitivity of locomotion to MK-801.

**Conclusions:**

These findings suggest that vitamin A depletion affect broad basal behavior and disrupt homeostasis maintaining capability in response to glutamate perturbation. We provide a useful animal model for assessing the role of vitamin A depletion in regulating animal behavior, and for detecting how neurotransmitter pathways might be involved in vitamin A depletion related behavioral abnormalities.

## Background

Vitamin A and its derivatives (retinoids) play indispensable roles in the development, maintenance and morphogenesis of the central nervous system [1,2]. By binding to their nuclear receptors(RARα, β, γ and RXRα, β, γ), retinoids induce or repress gene transcription by interacting with distinct promotor sequences in target genes [3]. Retinoid receptors are widely distributed in the cortex, hippocampus, nucleus accumbens, and other brain tissues [4]. During embryogenesis and early postnatal life, retinoic acid (the metabolic form of Vitamin A) facilitates nervous system development by guiding patterning and neuronal differentiation [1]. In rodents, the control of neural patterning and differentiation are disrupted when retinoic acid concentrations are lowered [5]. In mice, postnatal retinoic acid deficiency leads to significantly decreased neuronal differentiation within the granular cell layer of the dentate gyrus [6]. In vitro, blockage of retinoic acid signaling prevents glial-induced neuronal differentiation [7]. Retinoic acid also regulates neurite outgrowth [8]. Retinoic acid clearly has an important role in regulating nervous system processes.

Previous studies suggest the involvement of retinoid signaling in regulating the transcription of neurotransmitter receptors. The dopamine D2 receptor promoter contains a functional retinoic acid response element and is regulated by retinoic acid [9,10]. The dopamine D1 receptor is up-regulated by retinoids in the developing striatum [11]. In addition, glutamate NMDA receptors [12-14] and non-NMDA receptors [15] are induced by retinoids in vitro. It is well known that the dysfunction of dopamine and glutamate systems participate in the pathophysiological processes of affective disorders, and also regulate animal behavior. Pharmacological interference has been widely used to clarify the influences of glutamate/dopamine systems on psychosis behavior in animals and humans. MK-801 is a potent and selective antagonist of NMDA receptors, which has been successfully used to study the role of glutamate signaling alteration in animal behavior. In experimental rodents, MK-801 induces psychosis symptoms including locomotor hyperactivity, stereotypy and deficits in spatial memory [16-18]. MK-801 also increases the extracellular dopamine level and regulates the dopamine D1, D2, D3, and D4 receptor gene expression in the hippocampus in a complex manner [19].

Although one previous study [3] reported the role of vitamin A depletion in regulating animal motor abilities, the question of how vitamin A depletion mice respond to glutamate perturbation is still not clear. An understanding of this response would be valuable in explaining the influence of vitamin A depletion on homeostasis maintaining capability and in determining how glutamate/dopamine systems might be involved in vitamin A depletion regulated behavioral abnormalities. We therefore considered it of interest to explore the effect of vitamin A depletion on both basal mice behavior and the sensitivity of mice behavior to MK-801 interference.

In the current study, we measured the effect of vitamin A depletion on broad behavioral paradigms (motor abilities and pain sensitivity) under two conditions: prior to any injection and after the injection of MK-801, and found that vitamin A depletion induced mild changes in body weight and basal behavior and that vitamin A depletion altered the sensitivity of motor abilities and nociception to glutamate perturbation in mice.

## Methods

### Animal preparation

C57BL6 Jico inbred strain mice (Shanghai Laboratory Animal Center, Chinese Academy of Science, Shanghai, China; Warrant No. SCXK [Shanghai] 2007-0005) were housed at a constant temperature of 25 ± 2°C and 60% relatively humidity, on a 12 hour light-dark cycle (lights on at 7:00 h); food and water were available ad libitum. All experiments were conducted in accordance with the PRC national standards for laboratory animal quality and the Chinese guidelines for the care and use of laboratory animals.

For the generation of vitamin A depletion mice, successfully mated pregnant C57BL/6J mice were fed with a vitamin A free diet (D03102201) until the pups were weaned at the age of 3 weeks. Fifty six pups (male: female = 1:1) were selected randomly from 8 different litters, 28 of which (14 male, 14 female) were fed with a vitamin A free diet until they were sacrificed after the behavioral test. The other 28 pups (14 male, 14 female) were fed with a diet containing vitamin A 4,000 IU/kg (D06051001). The detailed composition of the vitamin A free diet (D03102201) and the normal diet (D06051001) are given in Additional file 1.

A separate group was housed and given vitamin A free food or normal food in the same manner as the previous group. Serums from 9-week mice were separated and stored at -70°C until analysis. The serums were analyzed for total retinol concentration using the high performance liquid chromatography (HPLC) method [3].

### Drugs

MK-801 was purchased from Sigma-Aldrich and dissolved in saline. As shown in a previous study [20], the injection of MK-801 (0.6 mg/kg, intraperitoneal [i.p.]) can induce hyperlocomotion, stereotypy and ataxia in mice. We selected this dose of MK-801 (0.6 mg/kg) to inject the mice and to observe subsequent behavior.

### Behavioral measures

The offspring (vitamin A depletion group: male n = 14, female n = 14; control group: male n = 14, female n = 14) were transferred to the behavior testing facilities at 9 weeks of age and allowed 3-8 days to acclimatize before behavioral testing. The individual pups were randomly selected from 8 different litters and used as statistical units instead of litters for the analysis of behavioral results. This design will not overestimate any effects (type 1 error) (See previous study [21]).

Two rounds of behavioral testing were conducted for all animals. In the first round test (prior to MK-801 injection), locomotion, rearing and tail flick activity were tested in mice with the two different diets. One day after the first round test, the second round test was conducted. Mice from each of the two diet groups were injected only once with MK-801 (0.6 mg/kg, intraperitoneal [i.p.]) prior to the series of behavioral tests. The same behavioral paradigms as in the first round, as well as stereotypy and ataxia, were tested. All behavior testing occurred during daytime hours (from 8:00 to 17:00).

### Locomotion and rearing

Locomotion measurement was assessed using automatic detection beam crossings in the Flex Field system (San Diego Instuments, San Diego, California, USA). Animals were placed in a 45 × 25 cm chamber (height 20 cm). Horizontal motion was detected by photobeams spaced at 5.0 cm lengthwise and 4.5 cm across, at a height of 2 cm. Rearing activity was measured by a second group of photodetectors mounted higher up, at 6 cm, at 2.5 cm intervals in the width direction. The recordings included the initial periods of adaptation to the novel environment. Locomotion and rearing were monitored for 3 hours. Data are presented as beam breaks per 5-min period unless otherwise indicated.

### Stereotypy and ataxia

Stereotypic behavior represents amounts of repetitive behavior, usually at the expense of locomotion or other natural behavior [22]. To measure stereotypic behavior, the animals in the measurement chambers were videotaped for 3 hour periods immediately after injection of MK-801 [20]. Stereotypy measurement was made for the first 2 min out of each 5 min time segment. Behavior that could be observed in normal, drug-naive animals, such as grooming or gnawing, were not scored as stereotypy unless they lasted longer than 3 s. Each videotape had to be rated by two independent scorers who were blind to the experimental conditions. Scorers were trained as previously reported [20] so as to assure rating reliability. Meanwhile, during the videotape analysis we also scored ataxia behavior as previously reported [20].

### Tail flick test

Thirty minutes after the locomotion test, we began the tail flick test using a Tail Flick Latency instrument (Stoelting, USA). A mouse was placed within a restraining tube with its tail protruding. The tail was placed on a level surface; a radiant heat was applied to the tail. Withdrawal latency was measured by the time taken for the mice to curl or flick the tail. The scores were based on the median of three tests, with 3-min intervals between each test.

### Statistical analysis

Statistical analysis was performed using SPSS14.0 for Windows. Means are shown with error bars indicating the standard error mean (SEM). Multivariate ANOVA was used to analyze the data for locomotion, rearing, tail flick latency, stereotypy and ataxia prior to and after MK-801 injection, and diet and sex were selected as fixed factors. Univariate analysis of variance was used to analyze the data of weight using diet and sex as fixed factors. Data transformation (natural logarithm transformation) was used for the data for rearing and tail flick latency to meet the homogeneity of variance and normality. Independent sample t-tests were also used where necessary.

## Results

### Serum levels of retinol

Serum retinol concentrations were significantly decreased in 9 week old vitamin A depletion mice compared to the control mice and were 32.5 ± 16.3 ng/ml in vitamin A depletion mice (n = 3) and 350.12 ± 53.48 ng/ml in control mice (n = 3) respectively. Values are shown as mean ± S.E.M; P < 0.01 (independent t-test).

### Body weight

Vitamin A depletion mice had significantly less body weight than the control mice at 9 weeks of age [F (1, 51) = 8.667, P = 0.005]. Male mice had significantly more body weight than female mice [F (1, 51) = 82.96, P < 0.001], but the interaction of diet and sex was not significant [F (1, 51) = 0.989, P > 0.05].

### Behavioral experiments

#### Locomotion

**Figure 1:**
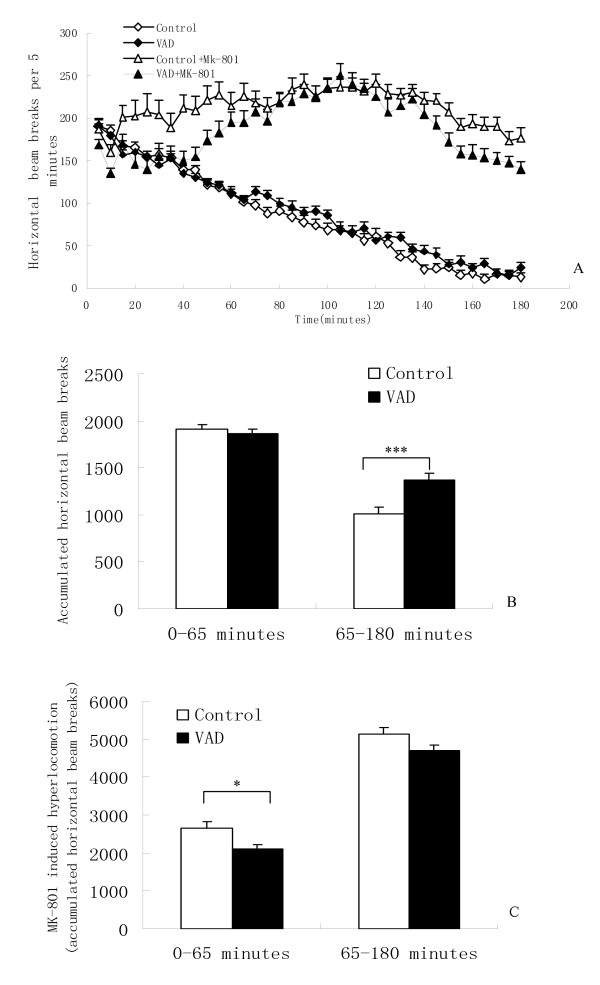
**Effects of vitamin A depletion (VAD) on locomotor activity and MK-801 (0.6 mg/Kg, i.p.) induced hyperlocomotion**. (A) Time course of locomotion activity. (B) Each column represents mean value ± SEM of accumulated locomotion performance. Vitamin A depletion mice showed significantly more locomotion from 65 to 180 minutes (***P < 0.001, t-test). There is no significant difference between vitamin A depletion and control mice in the first 65 minutes. (C) Vitamin A depletion mice had significantly less MK-801 induced hyperlocomotion in the first 65 minutes (*P < 0.05, t-test). There was no significant difference in MK-801 induced hyperlocomotion between vitamin A depletion and control mice from 65 to 180 minutes.

There was no main effect of diet [F (1, 47) = 2.172, P > 0.05] on accumulated horizontal beam breaks in the open field (Figure 1A). Vitamin A depletion mice showed no difference in locomotion (P > 0.05, t-test) compared to the control mice in the first 65 minutes of the test. However, vitamin A depletion mice showed significantly more cumulative locomotion from 65 to 180 minutes than the control mice (P < 0.001, t-test) (Figure 1B). After MK-801 injection, diet had a significant main effect [F (1, 46) = 12.28, P = 0.01] on locomotion. In particular, vitamin A depletion mice showed significantly less MK-801 induced hyperlocomotion (P < 0.05, t-test) (Figure 1C) than the control mice in the first 65 minutes. However, there was no significant difference in MK-801 induced hyperlocomotion between vitamin A depletion and control mice from 65 to 180 minutes (P > 0.05, t-test).

#### Rearing

**Figure 2:**
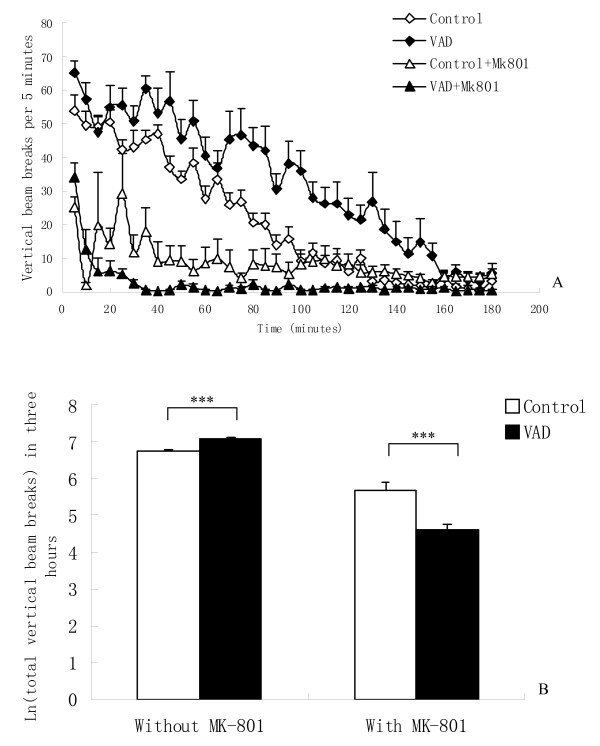
**Effects of vitamin A depletion (VAD) on rearing before and after MK-801 injection**. (A) Time course of vertical activities. (B) Total rearing activities in the first three hours. Each column represents mean ± SEM of ln (total rearing). Vitamin A depletion mice showed significantly more rearing than the control mice (***P < 0.001), but less rearing after MK-801 injection (***P < 0.001).

The natural logarithm of rearing data was used for data analysis in order to meet the homogeneity of variance and normality distribution. There was a significant main effect of diet [F (1, 47) = 18.63, P < 0.001] on rearing activities. In contrast to the stimulation of horizontal locomotion, rearing behavior has been shown to be significantly suppressed by MK-801 in normal mice [23]. After MK-801 injection, diet also proved to have a significant main effect [F (1, 46) = 14.787, P < 0.001] on rearing activities. Vitamin A depletion mice showed significantly increased rearing activity (P < 0.001), but after MK-801 injection vitamin A depletion induced significantly reduced rearing activity (P < 0.001) (Figure 2), suggesting that vitamin A depletion may amplify the suppression effect of MK-801 on rearing.

#### Stereotypy

**Figure 3:**
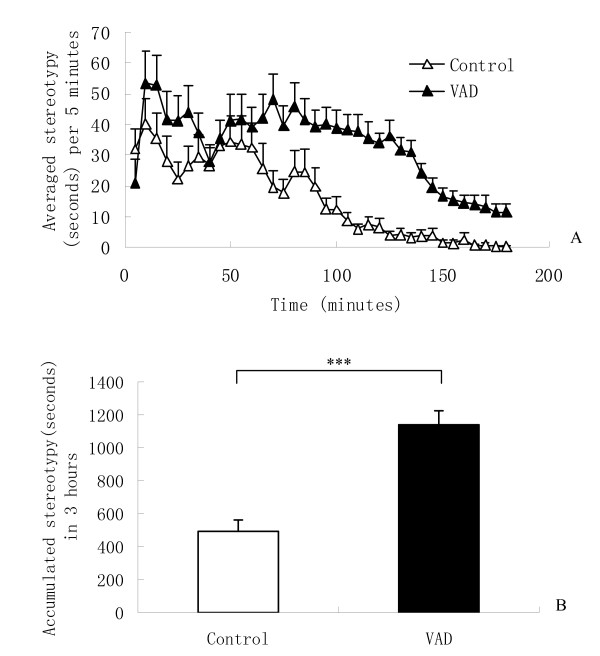
**Effects of vitamin A depletion (VAD) on MK-801 (0.6 mg/Kg, i.p.) induced stereotypy**. (A) Time course of MK-801 induced stereotypy in the first 3 hours. (B) Each column represents mean ± SEM of accumulated stereotypy during the first 3 hours. Vitamin A depletion mice showed significantly amplified MK-801 induced stereotypy (***P < 0.001) compared to the control mice.

The increase in stereotypy was induced after MK-801 injection (0.6 mg/kg, i.p.). There was a significant main effect of diet [F (1, 46) = 30.56, P < 0.001] on MK-801 induced stereotypy (Figure 3), indicating that vitamin A depletion induces hypersensitivity of stereotypy to MK-801.

#### Ataxia

**Figure 4:**
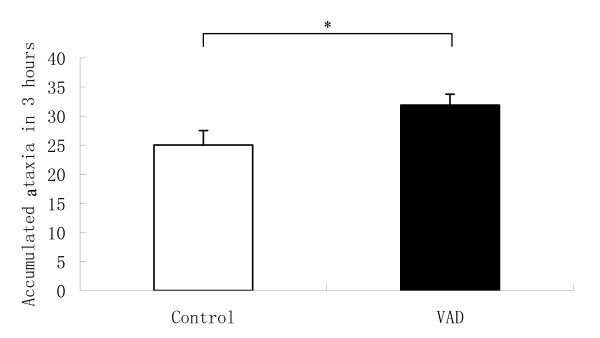
**Ataxia scores are shown as mean ± SEM**. There was a significant increase of ataxia in vitamin A depletion (VAD) mice (*P < 0.05).

ANOVA revealed a significant main effect of diet [F (1, 46) = 5.62, P = 0.022] on MK-801 induced ataxia (Figure 4) suggesting that vitamin A depletion results in hypersensitivity of ataxia to MK-801.

#### Tail flick latency

**Figure 5:**
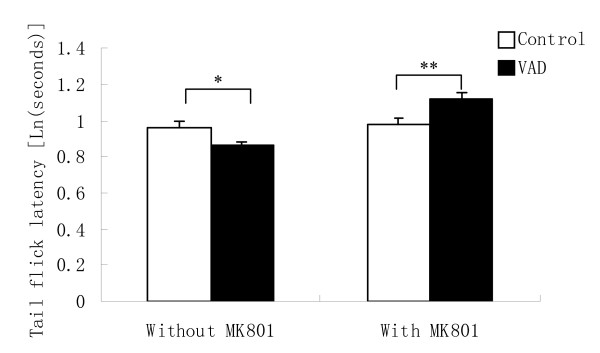
**Effects of vitamin A depletion (VAD) on tail flick latency without and with MK-801 injection**. Each column represents mean value ± SEM of mice tail flick latency [ln (seconds)]. Vitamin A depletion induced significantly shorter tail flick latency compared to the control mice (*P < 0.05). After MK-801 administration, vitamin A depletion mice displayed significantly longer tail flick latency than the controls (**P < 0.01).

The natural logarithm of tail flick latency data was used for ANOVA analysis in order to meet the homogeneity of variance and normality distribution. There was a significant main effect of diet [F (1, 47) = 6.747, P = 0.012] and sex [F (1, 47) = 15.534, P < 0.001] on tail flick latency. After MK-801 injection, diet also had a significant main effect [F (1, 47) = 13.99, P = 0.001] on tail flick latency. Prior to MK-801 injection, vitamin A depletion significantly reduced tail flick latency (P = 0.012) whereas after MK-801 injection vitamin A depletion mice showed significantly increased tail flick latency (P = 0.001) (Figure 5).

The body weight and behavioral information of both male and female mice are shown in Additional file2.

## Discussion

In the present study vitamin A depletion mice displayed subtle and discrete changes in body weight and basal behavior. It was noticed that vitamin A depletion mice had significantly lower body weight than the control mice, suggesting the general health of mice was adversely influenced by vitamin A depletion. The major behavioral findings in vitamin A depletion mice are: (1) enhanced locomotor activity; (2) increased rearing activity and (3) reduced tail flick latency.

Locomotor activity was used to measure the response to a novel environment. Vitamin A depletion mice displayed enhanced locomotor activity from 65 to 180 minutes of the test but not in the first 65 minutes. This suggests that the effect of vitamin A depletion on locomotor activity is subtle and dependent upon the detection period. In a study by Carta et al, postnatal vitamin A deficiency rats showed a significant hyperlocomotion in the first 20 minutes of the test [3]. This difference in time taken to show hyperlocomotion may be explained by the different animals used and the different methods used to generate the vitamin A depletion condition (we used vitamin A depletion mice, Carta et al used postnatal vitamin A deficiency rats). On the other hand, vitamin A depletion mice have also been shown to exhibit increased rearing activity, an exploratory behavior which reflects emotion and anxiety status [23]. This abnormality of rearing activity in vitamin A depletion mice could reflect the impairment of the ability to adapt to the external environment. Tail flick latency reflects the ability to respond to thermal nociception. In the assessment of pain sensitivity, less tail flick latency was found in vitamin A depletion mice, indicating that they had increased thermal pain sensitivity. To our knowledge, there have been no reports on the relationship between retinoids and nociception. The peripheral nociceptors or sensory neurons in the spinal cord may be regulated by retinoids.

To measure the interaction between vitamin A depletion and glutamate signaling, we also assessed the sensitivity of behavioral phenotypes to external influences (MK-801 injection) in the vitamin A depletion mice. These mice showed altered sensitivity of several types of behavior to MK-801, including stereotypy, ataxia, rearing, locomotion and tail flick latency, suggesting that homeostasis maintaining capability in vitamin A depletion mice is reduced.

It is well known that the injection of a moderate dose (0.6 kmg/kg) of MK-801 can induce hyperlocomotion, stereotypy and ataxia in mice. In the present study, the vitamin A depletion mice showed hypersensitivity of stereotypy and ataxia to the NMDA receptor antagonist (MK-801), indicating that the neural circuits which might be disrupted by vitamin A depletion are involved in the glutamate pathway. Furthermore, after MK-801 injection, the vitamin A depletion mice had significantly less hyperlocomotion than the control mice in the first 65 minutes, but showed no difference in hyperlocomotion from 65 to 180 minutes. It has been reported that hyperlocomotion and stereotypy are competing behaviors [20] and that increased stereotypy could inhibit hyperlocomotion. Although the vitamin A depletion mice showed hyposensitivity of locomotion to MK-801, this finding warrants further investigation, perhaps by using a different dose of MK-801 to test dose effectiveness. It has been found that rats with a neonatal lesion of the amygdale showed hypersensitivity of locomotion to low dose phencyclidine (PCP) (<2 mg/kg), while showing hyposensitivity of locomotion to high dose PCP (>2 mg/kg) [24]. Meanwhile, vitamin A depletion mice showed hypersensitivity of rearing activity to MK-801. MK-801 injection may induce severe ataxia in vitamin A depletion mice which could damage the motor ability of hind limbs and therefore decrease the rearing activity. In addition, vitamin A depletion mice showed significant increase in tail flick latency in response to MK-801, suggesting that vitamin A depletion exaggerates the vulnerability to external influences and might influence nociception sensitivity by regulating the pathway of peripheral NMDA receptor mediated responses to nociceptors [25,26].

The altered sensitivities of stereotypy, ataxia, rearing, locomotion and tail flick latency to MK-801, suggest the involvement of vitamin A depletion in regulating balancing ability in response to external glutamate perturbation. One of our previous studies found that inhibiting retinoic acid synthesis in astrocytes could result in altered proteins related to glutamate metabolism [27]. But until now, most of the evidence demonstrating the role of retinoids in regulating glutamate NMDA receptors (NR1, NR2) has come from in vitro studies [13]. The involvement of vitamin A in regulating glutamate pathway-related molecules warrants further studies, including testing the effect of vitamin A on glutamate receptors and glutamate metabolic enzymes in vivo. The motor phenotypes in vitamin A depletion mice also indicate alteration in the dopamine systems [28-30]. In previous studies, vitamin A signaling has been shown to play an important role in regulating the dopamine system. It has been shown that (1) reduction of retinoic acid influences the development and specifying of motor neurons [31], (2) the D2 dopaminergic receptor gene contains a functional retinoic acid receptor response element (RARE) that is activated by a RARα-RXRγ heterodimer [9], (3) Nurr-1, another heterodimeric partner of RXRs is expressed in developing dopaminergic neurons [32] and MK-801 can indirectly activate dopamine systems in the brain [33]. Therefore vitamin A depletion reduces the balancing abilities in response to external interference (MK-801 injection), probably by regulating the glutamate and dopamine pathway. Further studies of glutamate and dopamine pathway in different brain regions including hippocampus, cortex and striatum are required to clarify the mechanisms underlying altered sensitivity of stereotypy, ataxia, rearing, locomotion and tail flick latency to MK-801. Behavioral pharmacology studies using dopaminergic and glutamatergic agents are also valuable tools.

It has been found that behavioral changes in vitamin A depletion mice are related to psychosis symptoms. Enhanced locomotor ability and exaggerated sensitivity of stereotypy to MK-801 in vitamin A depletion mice are schizophrenia related symptoms [34,35] and are found in schizophrenia animal models [36-39]. However, hyposensitivity of locomotion to MK-801 does not correspond with the schizophrenia phenotype in animals. Stereotypy is also found in patients with mental retardation and autism spectrum disorders. Interestingly, vitamin A depletion mice displayed normal locomotion initially (from 0 to 65 minutes) but increased locomotion gradually (from 65 to 180 minutes), which is somewhat relevant to delayed hyperactivity in children with attention-deficit/hyperactivity disorder (ADHD) [40]. These findings add weight to the plausibility of the biological involvement of vitamin A cascade in the pathological processes of neuropsychiatric diseases.

### Limitations

The present study measured the response of vitamin A depletion mice and normal mice to the injection of single dose of MK-801 (0.6 mg/kg). The effect of MK-801 injection with different doses on motor behavior are deserved to be detected to reduce the possible bias caused by single dose injection. We proposed that glutamate system might be involved in vitamin A depletion related behavioral alteration. The detailed biochemical parameters should be measured to support that glutamate system is affected by vitamin A depletion, including the expression of glutamate receptors and the affinity for MK-801 on its receptors.

## Conclusions

In summary, we found that vitamin A depletion increased basal motor behavior and pain sensitivity in the mice, as well as altering the sensitivity of motor ability and nociception to external influences (glutamate perturbation). These results may reflect reduced capability of vitamin A depletion mice to maintain homeostasis. Our findings also indicate that vitamin A depletion may induce behavioral alterations by disturbing the glutamate/dopamine pathway. The present study therefore indicates that vitamin A depletion mice are a valuable animal model for studying the role of vitamin A in regulating a range of behavior in mice, and for investigating the involvement of vitamin A signaling in important neurotransmitters pathways such as glutamate signaling.

## Competing interests

The authors declare that they have no competing interests.

## Authors' contributions

ZM carried out HPLC experiments, data analysis and interpretation. ZBH, ZH, SJW and ZH conducted the behavioral experiments. ZZ assisted in data analysis. YL and JML revised the manuscript and provided suggestions. FGY provided experimental drugs. WCL and HL directed the overall project and coordinated data collection. All authors have read and approved the final manuscript.

## Supplementary Material

Additional file 1 **Rodent diet with 10 kcal% fat and modifications with or without added vitamin A**. The formula of diet with or without added vitamin A in the current study. Click here for file

Additional file 2 **Body weight and behavioral information in VAD and control mice**. Behavioral results in VAD and control mice were showed, including locomotion, rearing, tail flick latency, stereotype and ataxia with and without MK-801 injection. In addition, the body weight of VAD and control mice were also showed. Click here for file

## References

[B1] Maden M Retinoic acid in the development, regeneration and maintenance of the nervous system Nat Rev Neurosci 2007 8 755 65 10.1038/nrn2212 17882253

[B2] Lamantia AS Forebrain induction, retinoic acid, and vulnerability to schizophrenia: insights from molecular and genetic analysis in developing mice Biol Psychiatry 1999 46 19 30 10.1016/S0006-3223(99)00002-5 10394471

[B3] Carta M Stancampiano R Tronci E Collu M Usiello A Morelli M Fadda F Vitamin A deficiency induces motor impairments and striatal cholinergic dysfunction in rats Neuroscience 2006 139 1163 72 10.1016/j.neuroscience.2006.01.027 16530976

[B4] Krezel W Kastner P Chambon P Differential expression of retinoid receptors in the adult mouse central nervous system Neuroscience 1999 89 1291 300 10.1016/S0306-4522(98)00342-X 10362315

[B5] McCaffery PJ Adams J Maden M Rosa-Molinar E Too much of a good thing: retinoic acid as an endogenous regulator of neural differentiation and exogenous teratogen Eur J Neurosci 2003 18 457 72 10.1046/j.1460-9568.2003.02765.x 12911743

[B6] Jacobs S Lie DC DeCicco KL Shi Y DeLuca LM Gage FH Evans RM Retinoic acid is required early during adult neurogenesis in the dentate gyrus Proc Natl Acad Sci USA 2006 103 3902 7 10.1073/pnas.0511294103 16505366 PMC1450163

[B7] Környei Z Gócza E Rühl R Orsolits B Vörös E Szabó B Vágovits B Madarász E Astroglia-derived retinoic acid is a key factor in glia-induced neurogenesis FASEB J 2007 21 2496 509 10.1096/fj.06-7756com 17438145

[B8] Clagett-Dame M McNeill EM Muley PD Role of all-trans retinoic acid in neurite outgrowth and axonal elongation J Neurobiol 2006 66 739 56 10.1002/neu.20241 16688769

[B9] Samad TA Krezel W Chambon P Borrelli E Regulation of dopaminergic pathways by retinoids: activation of the D2 receptor promoter by members of the retinoic acid receptor-retinoid X receptor family Proc Natl Acad Sci USA 1997 94 14349 54 10.1073/pnas.94.26.14349 9405615 PMC24972

[B10] Farooqui SM Induction of adenylate cyclase sensitive dopamine D2-receptors in retinoic acid induced differentiated human neuroblastoma SHSY-5Y cells Life Sci 1994 55 1887 93 10.1016/0024-3205(94)00520-6 7990648

[B11] Wang HF Liu FC Regulation of multiple dopamine signal transduction molecules by retinoids in the developing striatum Neuroscience 2005 134 97 105 10.1016/j.neuroscience.2005.04.008 15939542

[B12] Kulikov AV Rzhaninova AA Goldshtein DV Boldyrev AA Expression of NMDA receptors in multipotent stromal cells of human adipose tissue under conditions of retinoic acid-induced differentiation Bull Exp Biol Med 2007 144 626 9 10.1007/s10517-007-0390-6 18642726

[B13] Pizzi M Boroni F Bianchetti A Moraitis C Sarnico I Benarese M Goffi F Valerio A Spano P Expression of functional NR1/NR2B-type NMDA receptors in neuronally differentiated SK-N-SH human cell line Eur J Neurosci 2002 16 2342 50 10.1046/j.1460-9568.2002.02403.x 12492429

[B14] Jelitai M Schlett K Varju P Eisel U Madarász E Regulated appearance of NMDA receptor subunits and channel functions during in vitro neuronal differentiation J Neurobiol 2002 51 54 65 10.1002/neu.10049 11920728

[B15] Younkin DP Tang CM Hardy M Reddy UR Shi QY Pleasure SJ Lee VM Pleasure D Inducible expression of neuronal glutamate receptor channels in the NT2 human cell line Proc Natl Acad Sci USA 1993 90 2174 8 10.1073/pnas.90.6.2174 7681588 PMC46048

[B16] Tricklebank MD Singh L Oles RJ Preston C Iversen SD The behavioural effects of MK-801: a comparison with antagonists acting non-competitively and competitively at the NMDA receptor Eur J Pharmacol 1989 167 127 35 10.1016/0014-2999(89)90754-1 2550253

[B17] Liljequist S Ossowska K Grabowska-Anden M Anden NE Effect of the NMDA receptor antagonist, MK-801, on locomotor activity and on the metabolism of dopamine in various brain areas of mice Eur J Pharmacol 1991 195 55 61 10.1016/0014-2999(91)90381-Y 1829683

[B18] Manahan-Vaughan D von Haebler D Winter C Juckel G Heinemann U A single application of MK801 causes symptoms of acute psychosis, deficits in spatial memory, and impairment of synaptic plasticity in rats Hippocampus 2008 18 125 34 10.1002/hipo.20367 17924525

[B19] Healy DJ Meador-Woodruff JH Dopamine receptor gene expression in hippocampus is differentially regulated by the NMDA receptor antagonist MK-801 Eur J Pharmacol 1996 306 257 64 10.1016/0014-2999(96)00204-X 8813639

[B20] Wu J Zou H Strong JA Yu J Zhou X Xie Q Zhao G Jin M Yu L Bimodal effects of MK-801 on locomotion and stereotypy in C57BL/6 mice Psychopharmacology (Berl) 2005 177 256 63 10.1007/s00213-004-1944-1 15290006

[B21] Per Eriksson Response to: Use of the Pup as the Statistical Unit in Developmental Neurotoxicity Studies: Overlooked Model or Poor Research Design? Toxicological Sciences 2008 103 411 413 10.1093/toxsci/kfn037 18463102

[B22] Qi C Zou H Zhang R Zhao G Jin M Yu L Age-related differential sensitivity to MK-801-induced locomotion and stereotypy in C57BL/6 mice Eur J Pharmacol 2008 580 161 8 10.1016/j.ejphar.2007.07.071 18053981 PMC2705961

[B23] Lever C Burton S O'Keefe J Rearing on hind legs, environmental novelty, and the hippocampal formation Rev Neurosci 2006 17 111 33 16703946 10.1515/revneuro.2006.17.1-2.111

[B24] Daenen EW Wolterink G Van Ree JM Hyperresponsiveness to phencyclidine in animals lesioned in the amygdala on day 7 of life. Implications for an animal model of schizophrenia Eur Neuropsychopharmacol 2003 13 273 9 10.1016/S0924-977X(03)00029-4 12888187

[B25] Du J Zhou S Coggeshall RE Carlton SM N-methyl-D-aspartate-induced excitation and sensitization of normal and inflamed nociceptors Neuroscience 2003 118 547 62 10.1016/S0306-4522(03)00009-5 12699789

[B26] Jang JH Kim DW Sang Nam T Se Paik K Leem JW Peripheral glutamate receptors contribute to mechanical hyperalgesia in a neuropathic pain model of the rat Neuroscience 2004 128 169 76 10.1016/j.neuroscience.2004.06.040 15450364

[B27] Zhang M Wan C Ji B Zhang Z Zhu H Tian N La Y Huang K Jiang L He G Gao L Zhao X Shi Y Huang G Feng G He L Proteome alteration of U251 human astrocytoma cell after inhibiting retinoic acid synthesis Mol Cell Biochem 2009 323 185 93 10.1007/s11010-008-9978-z 19089318

[B28] Chartoff EH Marck BT Matsumoto AM Dorsa DM Palmiter RD Induction of stereotypy in dopamine-deficient mice requires striatal D1 receptor activation Proc Natl Acad Sci USA 2001 98 10451 6 10.1073/pnas.181356498 11517332 PMC56981

[B29] Delis F Mitsacos A Giompres P Dopamine receptor and transporter levels are altered in the brain of Purkinje Cell Degeneration mutant mice Neuroscience 2004 125 255 68 10.1016/j.neuroscience.2004.01.020 15051164

[B30] Giros B Jaber M Jones SR Wightman RM Caron MG Hyperlocomotion and indifference to cocaine and amphetamine in mice lacking the dopamine transporter Nature 1996 379 606 12 10.1038/379606a0 8628395

[B31] Ji SJ Zhuang B Falco C Schneider A Schuster-Gossler K Gossler A Sockanathan S Mesodermal and neuronal retinoids regulate the induction and maintenance of limbinnervating spinal motor neurons Dev Biol 2006 297 249 61 10.1016/j.ydbio.2006.05.015 16781703

[B32] Zetterström RH Solomin L Jansson L Hoffer BJ Olson L Perlmann TRH Dopamine neuron agenesis in Nurr1-deficient mice Science 1997 276 248 50 10.1126/science.276.5310.248 9092472

[B33] Cook CD Newman JL Winfree JC Beardsley PM Modulation of the locomotor activating effects of the noncompetitive NMDA receptor antagonist MK801 by dopamine D2/3 receptor agonists in mice Pharmacol Biochem Behav 2004 77 309 18 10.1016/j.pbb.2003.11.002 14751459

[B34] Powell CM Miyakawa T Schizophrenia-relevant behavioral testing in rodent models: a uniquely human disorder? Biol Psychiatry 2006 59 1198 07 10.1016/j.biopsych.2006.05.008 16797265 PMC3928106

[B35] Bubeníková-Valesová V Horácek J Vrajová M Höschl C Models of schizophrenia in humans and animals based on inhibition of NMDA receptors Neurosci Biobehav Rev 2008 32 1014 23 10.1016/j.neubiorev.2008.03.012 18471877

[B36] Kesby JP Burne TH McGrath JJ Eyles DW Developmental vitamin D deficiency alters MK 801-induced hyperlocomotion in the adult rat: An animal model of schizophrenia Biol Psychiatry 2006 60 591 6 10.1016/j.biopsych.2006.02.033 16697353

[B37] Al-Amin HA Weinberger DR Lipska BK Exaggerated MK-801-induced motor hyperactivity in rats with the neonatal lesion of the ventral hippocampus Behav Pharmacol 2000 11 269 78 11103881 10.1097/00008877-200006000-00010

[B38] Mohn AR Gainetdinov RR Caron MG Koller BH Mice with reduced NMDA receptor expression display behaviors related to schizophrenia Cell 1999 98 427 436 10.1016/S0092-8674(00)81972-8 10481908

[B39] Rojas P Joodmardi E Hong Y Perlmann T Ogren SO Adult mice with reduced Nurr1 expression: an animal model for schizophrenia Mol Psychiatry 2007 12 756 66 10.1038/sj.mp.4001993 17457314

[B40] Sagvolden T Russell VA Aase H Johansen EB Farshbaf M Rodent models of attention-deficit/hyperactivity disorder Biol Psychiatry 2005 57 1239 1247 10.1016/j.biopsych.2005.02.002 15949994

