# Supplementary material for: Vitamin A depletion alters sensitivity of motor behavior to MK-801 in C57BL/6J mice
Source: Behav Brain Funct. 2010 Jan 22;6:7. doi: 10.1186/1744-9081-6-7 (PMC2832782; doi:10.1186/1744-9081-6-7)
Supplement: Additional file 1 — Rodent diet with 10 kcal% fat and modifications with or without added vitamin A. The formula of diet with or without added vitamin A in the current study. [file 1744-9081-6-7-S1.DOC]

Rodent Diet With 10 kcal% Fat and Modifications
 With or Without Added Vitamin A

| **Product #** | **D12450B** | |  | **D03102201** | | **D06051001** | |
| --- | --- | --- | --- | --- | --- | --- | --- |
| % | gm | *kcal* |  | gm | *kcal* | gm | *kcal* |
| Protein | 19.2 | *20* |  | 19.2 | *20* | 19.2 | *20* |
| Carbohydrate | 67.3 | *70* |  | 66.3 | *69* | 67.3 | *70* |
| Fat | 4.3 | *10* |  | 4.3 | *10* | 4.3 | *10* |
| Total |  | *100* |  |  | *99* |  | *100* |
| kcal/gm | 3.85 |  |  | 3.85 |  | 3.85 |  |
|  |  |  |  |  |  |  |  |
| **Ingredient** | **gm** | ***kcal*** |  | **gm** | ***kcal*** | **gm** | ***kcal*** |
| Casein, 80 Mesh | 200 | *800* |  | 0 | 0 | 0 | 0 |
| Casein, Alcohol Extracted | 0 | 0 |  | 200 | *800* | 200 | *800* |
| L-Cystine | 3 | *12* |  | 3 | *12* | 3 | *12* |
| Corn Starch | 315 | *1260* |  | 315 | *1260* | 315 | *1260* |
| Maltodextrin 10 | 35 | *140* |  | 35 | *140* | 35 | *140* |
| Sucrose | 350 | *1400* |  | 350 | *1400* | 350 | *1400* |
| Cellulose, BW200 | 50 | 0 |  | 50 | 0 | 50 | 0 |
| Soybean Oil | 25 | *225* |  | 0 | 0 | 0 | 0 |
| Lard | 20 | *180* |  | 0 | 0 | 0 | 0 |
| Cottonseed Oil | 0 | 0 |  | 45 | *405* | 45 | *405* |
| Mineral Mix S10026 | 10 | 0 |  | 10 | 0 | 10 | 0 |
| DiCalcium Phosphate | 13 | 0 |  | 13 | 0 | 13 | 0 |
| Calcium Carbonate | 5.5 | 0 |  | 5.5 | 0 | 5.5 | 0 |
| Potassium Citrate, 1 H2O | 16.5 | 0 |  | 16.5 | 0 | 16.5 | 0 |
| Vitamin Mix V10001(including Vitamin A) | 10 | *40* |  | 0 | 0 | 10 | *40* |
| V13001 (No added Vitamin A) | 0 | 0 |  | 10 | *40* | 0 | 0 |
| Choline Bitartrate | 2 | 0 |  | 2 | 0 | 2 | 0 |
| FD&C Yellow Dye #5 | 0.05 | 0 |  | 0 | 0 | 0 | 0 |
| FD&C Red Dye #40 | 0 | 0 |  | 0.05 | 0 | 0 | 0 |
| FD&C Blue Dye #1 | 0 | 0 |  | 0 | 0 | 0.05 | *0* |
|  |  |  |  |  |  |  |  |
| **Total** | **1055.05** | ***4057*** |  | **1055.05** | ***4057*** | **1055.05** | ***4057*** |

Formulated by Research Diets, Inc., 5/10/06
