# Supplementary material for: Vitamin A depletion alters sensitivity of motor behavior to MK-801 in C57BL/6J mice
Source: Behav Brain Funct. 2010 Jan 22;6:7. doi: 10.1186/1744-9081-6-7 (PMC2832782; doi:10.1186/1744-9081-6-7)
Supplement: Additional file 2 — Body weight and behavioral information in VAD and control mice. Behavioral results in VAD and control mice were showed, including locomotion, rearing, tail flick latency, stereotype and ataxia with and without MK-801 injection. In addition, the body weight of VAD and control mice were also showed. [file 1744-9081-6-7-S2.DOC]

Body weight and behavioral information in VAD and control mice

|  | Male (Mean±SEM) | | | Female (Mean±SEM) | | | Total (Mean±SEM) | | |
| --- | --- | --- | --- | --- | --- | --- | --- | --- | --- |
|  | Control | VAD | CN vs VN^d^ | Control | VAD | CN vs VN | Control | VAD | CN vs VN |
| **Body weight** ^e^ **(g)** | 22.5±0.34 | 21±0.29^*^ | 14vs14 | 18.7±0.54 | 18±0.24 | 14vs14 | 20.6±0.48 | 19.5±0.35^*^ | 28vs28 |
| **Open field** |  |  |  |  |  |  |  |  |  |
| Locomotion ^a^ | 2823±185 | 3228±183 | 14vs14 | 3222±152 | 3225±147 | 14vs13 | 3023±123 | 3227±116 | 28vs27 |
| Locomotion with MK-801 | 7647±239 | 6822±221^*^ | 14vs13 | 7610±463 | 6607±272 | 13vs13 | 7629±249 | 6715±173^*^ | 27vs26 |
| Rearing ^b^ | 754±47.2 | 1209±115^*^ | 14vs14 | 1053±63.8 | 1262±69.5^*^ | 12vs12 | 892±48.3 | 1230±68.8^*^ | 26vs26 |
| Rearing with MK-801 | 316±96.2 | 93.6±15.0^*^ | 14vs13 | 784±218 | 185±45.4^*^ | 13vs13 | 557±129 | 139±25.1^*^ | 27vs26 |
| Stereotypy (seconds) with MK-801^c^ | 369±69.9 | 1081±136^*^ | 14vs13 | 626±115 | 1199±96.1^*^ | 13vs13 | 493±69.5 | 1140±84.2^*^ | 27vs26 |
| Ataxia with MK-801^c^ | 26.4±3.23 | 28.5±2.41 | 14vs13 | 23.5±3.91 | 35.3±2.80^*^ | 13vs13 | 25.0±2.50 | 31.9±1.89^*^ | 27vs26 |
| **Tail flick test** |  |  |  |  |  |  |  |  |  |
| TFL (seconds) | 2.44±0.11 | 2.28±0.06 | 13vs14 | 2.84±0.11 | 2.50±0.08^*^ | 14vs13 | 2.64±0.09 | 2.39±0.05^*^ | 27vs27 |
| TFL with MK-801 | 2.54±0.10 | 3.25±0.18^*^ | 13vs13 | 2.87±0.22 | 2.96±0.14 | 13vs13 | 2.70±0.12 | 3.10±0.12^*^ | 26vs26 |

1. Locomotion is total horizontal beam breaks in the first three hours.
2. Rearing is total vertical beam breaks in three hours.
3. Accumulated stereotypy or ataxia is accessed in the first three hours.
4. CN represents the numbers of control mice, VN represents the numbers of VAD mice.
5. Body weight of mice with 9 weeks age.

* means P<0.05 (significance calculation was conducted after data transformation where necessary).
